# Draft Genome Sequence of *Commensalibacter papalotli* MX01, a Symbiont Identified from the Guts of Overwintering Monarch Butterflies

**DOI:** 10.1128/genomeA.00128-14

**Published:** 2014-03-06

**Authors:** Luis E. Servín-Garcidueñas, Andrés Sánchez-Quinto, Esperanza Martínez-Romero

**Affiliations:** Center for Genomic Sciences, Department of Ecological Genomics, National University of Mexico, Cuernavaca, Morelos, Mexico

## Abstract

We report the draft genome sequence of *Commensalibacter papalotli* strain MX01, isolated from the intestines of an overwintering monarch butterfly. The 2,332,652-bp AT-biased genome of *C. papalotli* MX01 is the smallest genome for a member of the *Acetobacteraceae* family and provides the first evidence of plasmids in *Commensalibacter*.

## GENOME ANNOUNCEMENT

Adult monarch butterflies feed on nectar that provides sugars and other nutrients. Monarch butterflies migrate to Mexican forests for overwintering. Overwintering monarchs reduce their metabolism and limit their feeding. Few studies have described gut bacteria in butterflies (1–8), and there are no studies on the molecular identification of symbionts from monarch butterflies.

*Commensalibacter* is a newly described genus of acetic acid bacteria (9). 16S rRNA gene sequences related to the *Commensalibacter* genus have been recovered from the guts of *Drosophila* species (9, 10), honey bees, and bumble bees (11, 12), as well as from *Heliconius erato* butterflies (1). The type strain *Commensalibacter intestini* A911 was isolated from *Drosophila* intestines (9), and its draft genome sequence has been reported (13). Here, we report the genome of a *Commensalibacter* symbiont isolated from a monarch butterfly.

A female monarch was collected in January 2010 from the Monarch Butterfly Biosphere Reserve in Mexico. Its gut contents were inoculated on Lee’s multi-differential agar (LMDA) medium (14) and were incubated at 25°C for 1 week. Colonies were identified by amplifying and sequencing 16S rRNA genes. Genomic DNA from *Commensalibacter papalotli* strain MX01 was isolated using the UltraClean microbial DNA kit (Mo-Bio Laboratories, Inc., Carlsbad, CA). The genome was sequenced using the Illumina HiSeq 2000 platform with a paired-end library. A total of 49,933,356 100-bp reads were generated. Twelve contigs with a sequence length of 2,332,652 bp were assembled *de novo* using Velvet version 1.2.10 (15). The genome coverage reached 2,117.64-fold, and the N_50_ length is 1,547,573 bp. Genome annotation was performed by the NCBI Prokaryotic Genomes Annotation Pipeline version 2.0 (https://www.ncbi.nlm.nih.gov/genome/annotation_prok/). Average nucleotide identity (ANI) values were calculated as previously proposed (16) using the ANI calculator from the Kostas lab (http://enve-omics.ce.gatech.edu/ani/).

The genome of strain MX01 has a G+C content of 36.66% and consists of a chromosome and two complete circular plasmids. The chromosome is contained in 10 contigs, with a length of 2,310,436 bp. Plasmid pA is 15,425 bp and codes for a VapBC toxin-antitoxin system, plasmid replication and partitioning (encoded by *repA*, *parA*, and *parB*), a putative glycosyltransferase, a putative carbohydrate-selective porin (OprB), and proteins for iron acquisition and metabolism, including a transporter, a lipoprotein, a permease, and a polyferredoxin. Plasmid pB is 6,791 bp and codes for a RepB protein, an integrase, and hypothetical proteins. A total of 2,105 genes were predicted, including 2,060 protein-coding genes, 3 rRNAs, and 43 tRNAs. Function predictions were assigned to 1,718 protein-coding genes.

The 16S rRNA gene from strain MX01 has 98% sequence identity with the corresponding gene of *C. intestini* A911^T^. Genome comparisons between strains MX01 and A911^T^ revealed an ANI value of 82.95%. An ANI boundary of 95 to 96% has been useful for taxonomically circumscribing prokaryotic species (17). Strain MX01 then corresponds to a novel *Commensalibacter* species. The name *C. papalotli* MX01 is proposed. The word “papalotl” means butterfly in the Mexican Náhuatl language. A detailed characterization of *C. papalotli* MX01 is currently in progress.

### Nucleotide sequence accession numbers.

This whole-genome shotgun project has been deposited at DDBJ/EMBL/GenBank under the accession no. ATSX00000000. The version described in this paper is version ATSX01000000.
